# The SUMMIT Study: Utilising a written ‘Next Steps’ information booklet to prepare participants for potential lung cancer screening results and follow-up

**DOI:** 10.1016/j.lungcan.2022.12.006

**Published:** 2022-12-24

**Authors:** Amyn Bhamani, Carolyn Horst, Fanta Bojang, Samantha L Quaife, Jennifer L Dickson, Sophie Tisi, Helen Hall, Priyam Verghese, Andrew Creamer, Ruth Prendecki, John McCabe, Kylie Gyertson, Vicky Bowyer, Ethaar El-Emir, Alice Cotton, Simranjit Mehta, Claire Levermore, Anne-Marie Mullin, Jonathan Teague, Laura Farrelly, Arjun Nair, Anand Devaraj, Allan Hackshaw, SUMMIT consortium members, Sam M Janes, Jennifer L Dickson, Carolyn Horst, Sophie Tisi, Helen Hall, Priyam Verghese, Andrew Creamer, Thomas Callender, Ruth Prendecki, Amyn Bhamani, Mamta Ruparel, Allan Hackshaw, Laura Farrelly, Jon Teague, Anne-Marie Mullin, Kitty Chan, Rachael Sarpong, Malavika Suresh, Samantha L Quaife, Arjun Nair, Anand Devaraj, Kylie Gyertson, Vicky Bowyer, Ethaar El-Emir, Judy Airebamen, Alice Cotton, Kaylene Phua, Elodie Murali, Simranjit Mehta, Janine Zylstra, Karen Parry-Billings, Columbus Ife, April Neville, Paul Robinson, Laura Green, Zahra Hanif, Helen Kiconco, Ricardo McEwen, Dominique Arancon, Nicholas Beech, Derya Ovayolu, Christine Hosein, Sylvia Patricia Enes, Qin April Neville, Jane Rowlands, Aashna Samson, Urja Patel, Fahmida Hoque, Hina Pervez, Sofia Nnorom, Moksud Miah, Julian McKee, Mark Clark, Jeannie Eng, Fanta Bojang, Claire Levermore, Anant Patel, Sara Lock, Rajesh Banka, Angshu Bhowmik, Ugo Ekeowa, Zaheer Mangera, William M Ricketts, Neal Navani, Terry O’Shaughnessy, Charlotte Cash, Magali Taylor, Samanjit Hare, Tunku Aziz, Stephen Ellis, Anthony Edey, Graham Robinson, Alberto Villanueva, Hasti Robbie, Elena Stefan, Charlie Sayer, Nick Screaton, Navinah Nundlall, Lyndsey Gallagher, Andrew Crossingham, Thea Buchan, Tanita Limani, Kate Gowers, Kate Davies, John McCabe, Joseph Jacob, Karen Sennett, Tania Anastasiadis, Andrew Perugia, James Rusius, Sam M Janes

**Affiliations:** 1 Lungs For Living Research Centre, UCL Respiratory, University College London, London; 2 CRUK & UCL Cancer Trials Centre, University College London, London; 3 Centre for Prevention, Detection and Diagnosis, Wolfson Institute of Population Health, Barts and The London School of Medicine and Dentistry, Queen Mary University of London, London; 4 University College London Hospitals NHS Foundation Trust, London; 5 Royal Brompton and Harefield NHS Foundation Trust, London; 6 National Heart and Lung Institute, Imperial College, London; 7 Royal Free London NHS Foundation Trust, London; 8 Whittington Health NHS Trust, London; 9 Barking, Havering and Redbridge University Hospitals NHS Trust, Essex; 10 Homerton University Hospital Foundation Trust, London; 11 The Princess Alexandra Hospital NHS Trust, Essex; 12 North Middlesex University Hospital NHS Trust, London; 13 Barts Health NHS Trust, London; 14 North Bristol NHS Trust, Bristol; 15 Royal United Hospitals Bath NHS Foundation Trust, Bath; 16 Surrey and Sussex Healthcare NHS Trust, Surrey; 17 King’s College Hospital NHS Foundation Trust, London; 18 The Princess Alexandra Hospital NHS Trust, London; 19 University Hospitals Sussex NHS Foundation Trust, Sussex; 20 Royal Papworth Hospital NHS Foundation Trust, Cambridge; 21 Killick Street Health Centre, London; 22 Tower Hamlets Clinical Commissioning Group, London; 23 Noclor Research Support, London; 24 Centre for Medical Image Computing (CMIC), London; a Lungs for Living Research Centre, UCL Respiratory, University College London, London, UK; b University College London Hospitals NHS Foundation Trust, London, UK; c Centre for Prevention, Detection and Diagnosis, Wolfson Institute of Population Health, Barts and The London School of Medicine and Dentistry, Queen Mary University of London, London, UK; d Cancer Research UK and UCL Cancer Trials Centre, University College London, London, UK; e Royal Brompton and Harefield NHS Trust, London, UK; f National Heart and Lung Institute, Imperial College London, London, UK

**Keywords:** Lung Cancer Screening, Pulmonary Nodule, Result Communication, LDCT

## Abstract

**Objectives:**

Low-Dose Computed Tomography (LDCT) screening for lung cancer can result in several potential outcomes of varying significance. Communication methods used in Lung Cancer Screening (LCS) programmes must, therefore, ensure that participants are prepared for the range of possible results and follow-up. Here, we assess perceptions of a written preparatory information booklet provided to participants in a large LCS cohort designed to convey this information.

**Materials and Methods:**

All participants in the SUMMIT Study (NCT03934866) were provided with a results preparation information booklet, entitled ‘The SUMMIT Study: Next Steps’ at their baseline appointment which outlined potential results, their significance, and timelines for follow up. Results from the LDCT scan and Lung Health Check were subsequently sent by letter. Perceptions of this booklet were assessed among participants with indeterminate pulmonary findings when they attended a face-to-face appointment immediately before their three-month interval scan. Specifically, questions assessed the perceived usefulness of the booklet and the amount of information contained in it.

**Results:**

70.1% (n = 1,412/2,014) participants remembered receiving the booklet at their appointment. Of these participants, 72.0% (n = 1,017/1,412) found it quite or very useful and 68.0% (n = 960/1,412) reported that it contained the right amount of information. Older participants, those from the least deprived socioeconomic quintile and those of Black ethnicity were less likely to report finding the booklet either quite or very useful, or that it contained the right amount of information. Participants who remembered receiving the booklet were more likely to be satisfied with the process of results communication by letter.

**Conclusion:**

Providing written information that prepares participants for possible LDCT results and their significance appears to be a useful resource and a helpful adjunct to a written method of results communication for large scale LCS programmes.

## 1 Introduction

In addition to suspicious lesions needing urgent MDT assessment, Low-Dose Computed Tomography (LDCT) screening for lung cancer often identifies incidental and indeterminate findings which require either primary care follow-up or interval imaging. In UK based Lung Cancer Screening (LCS) programmes, up to 24% of participants have indeterminate pulmonary findings requiring a three-month follow up LDCT scan [1–4]. While the majority of these are pulmonary nodules, up to 12% of participants require follow up for other ‘non-nodule’ findings such as consolidation [1].

Informed decision-making about LCS should include information about the different potential LDCT results and further testing. However, this information may not hold much significance until the individual receives their respective LDCT result at which point it may be difficult to recall. Identifying methods which prepare and communicate the different results to ensure participants understand the meaning of their result at the point they receive it, as well as the relevant next steps, is imperative. A brief preparatory information booklet provided at the time of the LDCT scan may be one such method.

The SUMMIT Study (NCT03934866) is a prospective observational cohort study which aims to assess the implementation of LDCT screening for lung cancer in a high-risk population in North Central and East London and validate a multi-cancer early detection blood test. A brief written ‘Next Steps’ booklet designed specifically for the study was provided to participants at their baseline Lung Health Check (LHC) to prepare them for the possible LDCT results, how these would be conveyed and the types of follow-up that could be expected.

We have previously reported high participant satisfaction with the reporting of indeterminate pulmonary nodule results by letter [5]. Here, we present analysis of participants’ perceptions of the ‘Next Steps’ booklet to explore its utility as an adjunct to a written method of results communication in a large LCS cohort.

## 2 Materials and methods

Individuals aged 55–77 years and recorded as smokers in the past 20 years were identified from participating primary care practices and invited to undergo eligibility assessment for LCS via participation in the SUMMIT Study. The final step in this process involved attendance at a face-to-face LHC appointment [6]. All individuals participating in the study were given a booklet entitled ‘The SUMMIT Study: Next Steps’ at the end of this appointment. A Low-Dose Computed Tomography (LDCT) scan was carried out following this.

The booklet was developed using a multidisciplinary approach with input from specialists in psychology, respiratory medicine, and radiology. Several rounds of patient and public advisory group input were undertaken before a final version was approved to ensure that the information it contained was clear, concise and jargon free.

The booklet provided information about the types of LDCT results that could be expected, what these results mean, and a timeline for follow-up depending on the scan result (Supplementary Fig. 1). Information on lung nodules, the significance of abnormal results, smoking cessation advice and contact details for smoking cessation programmes was also included, along with contact details for the study team should participants have wished to obtain information on their results prior to their next appointment.

**Fig. 1:**
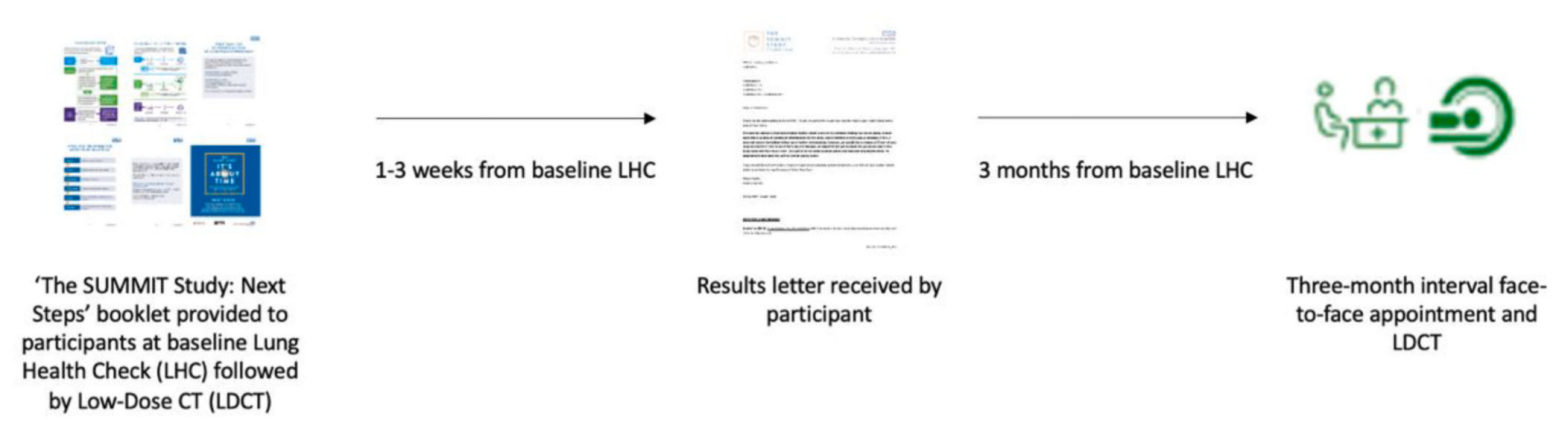
Timelines for participants in the SUMMIT Study undergoing three-month interval LDCT scans for indeterminate pulmonary findings.

Participants with indeterminate pulmonary findings requiring three-month follow up LDCT were informed of their results by letter (Fig. 1). When they attended for their follow-up scan, they were also given a face-to-face appointment with a research nurse or clinical trials practitioner during which they were verbally asked whether they remembered receiving ‘The SUMMIT Study: Next Steps’ booklet at their baseline visit. Those that remembered were asked to provide their opinion on the amount and usefulness of the information the booklet contained about the different types of LDCT results. For both questions, participants were provided with a range of options for response (shown in Table 2). Study team members conducting the appointment were not advised to give any specific prompts or reminders and if participants did not recall receiving the booklet, this was documented and no further questions on the subject were asked. Staff training was standardised and monitored for consistency.

At the same appointment, participants were also verbally asked to report their satisfaction with the written method of results communication used in the study and their preferred method. A range of options for response were provided. These have previously been published [5]. Finally, participants were asked if they had consulted primary care to discuss their LDCT results further and if they had any questions about their results letter. Responses to these questions were recorded as a binary outcome. The type of questions asked at this visit were also recorded using a pre-defined list [5]. More than one option could be selected, including ‘other,’ which was followed by a free text box for further documentation, if needed.

We reviewed records for participants who had indeterminate pulmonary findings detected on their baseline LDCT scan and attended for a three-month interval Lung Health Check (LHC) appointment and LDCT between 18th July 2019 and 10th August 2021.

The primary objectives were to assess the proportion of individuals who found the booklet useful and its impact on participant reported satisfaction with the process of results communication by letter. Secondary objectives included assessing participant perception of the amount of information in the booklet, its impact on the number of participants asking questions of the study team at the three-month appointment visit and its effect on primary care consultations to discuss LDCT results.

Univariable and multivariable binary logistic regression analyses were used to explore demographic and smoking characteristics of participants that reported finding the booklet quite or very useful, and those reporting that it contained the right amount of information. Multivariate models were adjusted for factors known to influence healthcare (including LCS) uptake including gender, age, socioeconomic deprivation, smoking status, highest education level and ethnicity. Chi-square test was used to compare responses between participants who did and did not remember receiving the booklet. A p-value of <0.05 was considered significant.

## 3 Results

13,035 individuals participated in the SUMMIT Study. 2,094 were initially invited for a three-month interval LHC appointment and 2,014 attended. 70.1% (n = 1,412/2,014) remembered receiving ‘The SUMMIT Study: Next Steps’ booklet at their baseline appointment. 57.9% (n = 818/1,412) of these were male and the mean age was 66.2 years (SD 5.98). 59.8% (n = 844/1,412) were from the two most deprived national socioeconomic quintiles and half (49.9%, n = 705/1,412) were current smokers. Of the 602 participants who did not remember receiving the booklet, 61.3% (n = 369/602) were male and the mean age was 67.4 years (SD 6.10). A larger proportion (66.7%, n = 401/602) were from the two most deprived national socioeconomic quintiles. 47.7% (n = 287/602) were current smokers (Table 1).

Of those that remembered receiving ‘The SUMMIT Study: Next Steps’ booklet, 72.0% (n = 1,017/1,412) found it either quite or very useful and only 0.8% (n = 11/1,412) found it not at all useful. 68.0% (n = 960/ 1,412) reported that the booklet contained just the right amount of information, with 1.0% (n = 14/1,412) and 1.2% (n = 17/1,412) reporting that it contained too much and not enough information respectively (Table 2).

Logistic regression analysis showed that participants aged ≥75 years were less likely to report finding the booklet quite or very useful (aOR 0.492; 95% CI: 0.307 – 0.789, p = 0.003). Similarly, participants from the least deprived socioeconomic quintile (IMD 5: aOR 0.472; 95% CI: 0.279 – 0.800, p = 0.005) and those of Black ethnicity (aOR 0.400; 95% CI: 0.173 – 0.921, p = 0.031) were less likely to report finding the booklet quite or very useful (Table 3).

Participants aged ≥70 years (70–74: aOR 0.684; 95% CI 0.469–0.997, p = 0.048; ≥75 aOR 0.558; 95% CI: 0.352 – 0.884, p = 0.013), those from the least deprived socioeconomic quintile (IMD 5: aOR 0.470; 95 %CI: 0.281 – 0.786, p = 0.004) and those of Black ethnicity (aOR 0.386; 95% CI: 0.172 – 0.864, p = 0.021) were less likely to report that the booklet contained just the right amount of information (Table 4).

No statistical associations were identified across gender, smoking status, or highest level of education.

Participants who remembered receiving the booklet were more likely to report satisfaction with the process of receiving results by letter compared with those that did not remember receiving the booklet (84.6%, n = 1,195/1,412 vs 78.6%, n = 473/602; p = 0.001). 79.5% (n = 1,122/1,412) of those that remembered receiving the booklet reported that their results letter contained just the right amount of information compared with 72.1% (n = 434/602) of those that did not (p < 0.001) (Table 5).

14.2% (n = 200/1,412) of participants who remembered receiving the booklet had discussed their results letter with their primary care doctor compared to 11.5% (n = 69/602) of those that did not (p = 0.103). 45.0% (n = 635/1,412) of those that remembered receiving the booklet asked questions of the study team at the three-month LHC compared with 37.2% (n = 224/602) of those that did not (p = 0.001). Participants’ preferred method of communication did not vary between the two groups.

## 4 Discussion

‘The SUMMIT Study: Next Steps’ booklet was well received by participants who underwent three-month interval LDCT imaging for indeterminate pulmonary findings. Of those who remembered receiving the booklet, most reported that they found it either quite or very useful (72.0%), and that it contained the right amount of information (68.0%). <1 % of participants reported finding the booklet not at all useful.

Participants who remembered receiving the booklet were more likely to report both satisfaction with the process of receiving results by letter and that the letter contained the right amount of information, suggesting that the booklet was useful in preparing participants for their LDCT result.

Older participants, those living in areas categorised within the least socioeconomically deprived quintile nationally and those of Black ethnicity were less likely to report finding the booklet quite or very useful, or that it contained the right amount of information.

Interestingly, remembering receiving the booklet was not associated with reduced primary care consultations to discuss results letters or the proportion of participants that asked questions of the study team at the follow-up LHC visit. Rather, participants who remembered receiving the booklet were more likely to ask questions of the study team at their follow-up visit compared with those that did not. One explanation for this may be that participants who remembered receiving the booklet did so because they are more likely to seek health information and so, are also more likely to ask questions about their result when given the opportunity. The booklet may also have helped them to prepare questions for their appointment.

As the number of participants who reported that the booklet was not useful, or contained insufficient or too much information was small, demographic analysis of these participants did not reach statistical significance. Larger scale studies would therefore be needed to quantify these perceptions to help inform more targeted preparatory information booklets to enhance the quality of communication for large scale LCS programmes in the future. Further qualitative research incorporating Patient and Public Involvement (PPI) focus groups, think aloud interviews and co-design [7,8] may also be beneficial.

A limitation of this study is the fact that a relatively large proportion of participants (29.9%) did not remember receiving the booklet. Although study team members conducting follow-up appointments were not advised to provide specific prompts or reminders to these participants, it is possible that some practitioners who had a greater knowledge of the booklet than others would prompt more, plausibly introducing a source of bias. Additionally, while potentially more memorable presentation modalities such as audio and video recordings have been shown to promote the recall of health-related information [9,10], the effectiveness of such modalities will need to be weighed up against the cost and logistical challenges associated with their development and distribution.

Delivering LCS on a population basis requires resource efficient communication methods. We have previously reported that a written method of results communication is satisfactory for most individuals found to have indeterminate pulmonary nodules on LDCT [5]. Here, we show that participants who remembered receiving ‘The SUMMIT Study: Next Steps’ booklet were more likely to report satisfaction with the communication of pulmonary nodule results by letter, suggesting that similar participant information booklets are a potentially useful preparatory adjunct to a written method of results communication for large scale LCS programmes.

## Supplementary Material

Supplementary data

## Figures and Tables

**Table 1:** Demographics of participants attending three-month interval LHC appointment who remembered receiving the ‘Next Steps’ booklet compared to those who did not.

	Participants who remembered receiving the booklet (n = 1,412)		Participants who did not remember receiving the booklet (n = 602)		p-value
	Frequency (n)	Percentage (%)	Frequency (n)	Percentage (%)	
Gender *					0.160
Female	594	42.1	233	38.7	
Male	818	57.9	369	61.3	
Mean age ^†^, (SD)					**<0.001**
	66.2 (SD 5.98)	–	67.4 (SD 6.10)	–	
Age ^†^ groups					**0.005**
55–59	228	16.1	75	12.5	
60–64	349	24.7	131	21.8	
65–69	378	26.8	153	25.4	
70–74	319	22.6	159	26.4	
≥75	138	9.8	84	14.0	
National Index of Multiple Deprivation (IMD)*					**0.015**
Quintile 1 (most deprived)	446	31.6	202	33.6	
Quintile 2	398	28.2	199	33.1	
Quintile 3	245	17.4	99	16.4	
Quintile 4	240	17.0	72	12.0	
Quintile 5 (least deprived)	76	5.4	25	4.2	
Missing	7	0.5	5	0.8	
Smoking status^‡^					0.354
Current smoker	705	49.9	287	47.7	
Former smoker	707	50.1	315	52.3	
Ethnicity^‡^					0.989
Asian	74	5.2	34	5.6	
Black	51	3.6	22	3.7	
Mixed	26	1.8	12	2.0	
Other	40	2.8	18	3.0	
White	1,210	85.7	507	84.2	
Missing	11	0.8	9	1.5	

* From primary care record

† Age at time of three-month interval LHC appointment

‡ Self-reported by participant at baseline (Y0) LHC.

**Table 2:** Participant reported perception of how much information was included in ‘The SUMMIT Study: Next Steps’ booklet and how useful this information was.

	Frequency (n)	%
In your opinion, how much information did the next steps booklet contain about the different types of low dose CT results?		
Too much information	14	1.0
Just the right amount of information	960	68.0
Not enough information	17	1.2
Can’t remember	421	29.8
How useful did you find the information about the different types of low dose CT results?		
Not at all useful	11	0.8
Quite useful	648	45.9
Very useful	369	26.1
Can’t remember	384	27.2

**Table 3:** Univariate and multivariate binary logistic regression analysis assessing demographic and smoking characteristics of individuals who reported that the information contained in the ‘Next Steps’ booklet was quite or very useful (n = 1,017/1,412).

	Frequency (n)	Percentage (%)	Univariate analysis		Multivariate analysis	
			Unadjusted odds ratio (OR), 95 % CI	p-value	Adjusted odds ratio (aOR), 95 % CI	p-value
Gender						
Female	431	42.4	1.00	–	1.00	–
Male	586	57.6	0.955 (0.755 – 1.209)	0.704	0.904 (0.708 – 1.155)	0.420
Age groups						
55–59	170	16.7	1.00	–	1.00	–
60–64	266	26.2	1.093 (0.743 – 1.610)	0.651	1.114 (0.751 – 1.654)	0.591
65–69	287	28.2	1.076 (0.736 – 1.574)	0.706	1.069 (0.724 – 1.578)	0.737
70–74	214	21.0	0.695 (0.476 – 1.015)	0.060	0.722 (0.487 – 1.071)	0.105
≥75	80	7.9	0.471 (0.300 – 0.739)	**0.001**	0.492 (0.307 – 0.789)	**0.003**
National Index of Multiple Deprivation (IMD) rank					
Quintile 1 (most deprived)	335	32.9	1.00	–	1.00	–
Quintile 2	285	28.0	1.093 (0.743 – 1.610)	0.651	0.815 (0.595 – 1.117)	0.203
Quintile 3	171	16.8	1.076 (0.736 – 1.574)	0.706	0.750 (0.522 – 1.078)	0.120
Quintile 4	176	17.3	0.695 (0.476 – 1.015)	0.060	0.880 (0.606– 1.279)	0.503
Quintile 5 (least deprived)	45	4.4	0.471 (0.300 – 0.739)	**0.001**	0.472 (0.279 – 0.800)	**0.005**
Missing	5	0.5	–	–	–	–
Smoking status (at baseline LHC)						
Former smoker	511	50.2	1.00	–	1.00	–
Current smoker	506	49.8	0.975 (0.773 – 1.230)	0.833	0.890 (0.696 – 1.138)	0.354
Highest level of education						
Finished school at or before 15	398	39.1	1.00	–	1.00	–
O-levels or equivalent	250	24.6	1.101 (0.823 – 1.473)	0.518	1.071 (0.792 – 1.448)	0.656
A-levels or equivalent	109	10.7	1.310 (0.867 – 1.980)	0.200	1.335 (0.865 – 2.060)	0.192
Further education but not degree	87	8.6	1.290 (0.821 – 2.025)	0.269	1.270 (0.794 – 2.032)	0.319
Bachelors or equivalent	121	11.9	1.454 (0.967 – 2.188)	0.072	1.395 (0.910 – 2.137)	0.127
Higher degree – Masters or PhD	52	5.1	1.779 (0.945 – 3.350)	0.075	1.752 (0.917 – 3.347)	0.089
Ethnicity						
Asian	59	5.8	1.00	–	1.00	–
Black	33	3.2	0.466 (0.208 – 1.044)	0.064	0.400 (0.173 – 0.921)	**0.031**
Mixed	20	2.0	0.847 (0.290 – 2.481)	0.763	0.647 (0.215 – 1.946)	0.438
Other	28	2.8	0.593 (0.245 – 1.434)	0.246	0.491 (0.197 – 1.223)	0.127
White	867	85.3	0.643 (0.360 – 1.148)	0.135	0.622 (0.337 – 1.148)	0.129
Missing	10	1.0	–	–	–	–

**Table 4:** Univariate and multivariate binary logistic regression analysis assessing demographic and smoking characteristics of individuals who reported that the ‘Next Steps’ booklet contained just the right amount of information (n = 960/1,412).

	Frequency (n)	Percentage (%)	Univariate analysis		Multivariate analysis	
			Unadjusted odds ratio (OR), 95 % CI	p-value	Adjusted odds ratio (aOR), 95 % CI	p-value
Gender					
Female	406	42.3	1.00	–	1.00	–
Male	554	57.7	0.974 (0.775 – 1.219)	0.804	0.915 (0.723 – 1.158)	0.460
Age groups					
55–59	161	16.8	1.00	–	1.00	–
60–64	249	25.9	1.036 (0.718 – 1.496)	0.850	1.064 (0.732 – 1.548)	0.745
65–69	279	29.1	1.173 (0.814 – 1.691)	0.393	1.189 (0.817 – 1.729)	0.366
70–74	194	20.2	0.646 (0.449 – 0.928)	**0.018**	0.684 (0.469 – 0.997)	**0.048**
≥75	77	8.0	0.525 (0.338 – 0.816)	**0.004**	0.558 (0.352 – 0.884)	**0.013**
IMD rank					
Quintile 1 (most deprived)	319	33.2	1.00	–	1.00	–
Quintile 2	269	28.0	0.830 (0.619 – 1.114)	0.214	0.818 (0.605 – 1.107)	0.194
Quintile 3	163	17.0	0.791 (0.566 – 1.107)	0.172	0.789 (0.556 – 1.120)	0.185
Quintile 4	163	17.0	0.843 (0.600 – 1.184)	0.324	0.827 (0.580– 1.181)	0.297
Quintile 5 (least deprived)	41	4.3	0.466 (0.284 – 0.766)	**0.003**	0.470 (0.281 – 0.786)	**0.004**
Missing	5	0.5	–	–	–	–
Smoking status (at baseline LHC)					
Former smoker	477	49.7	1.00	–	1.00	–
Current smoker	483	50.3	1.049 (0.839 – 1.312)	0.657	0.964 (0.761 – 1.220)	0.758
Highest level of education					
Finished school at or before 15	375	39.1	1.00	–	1.00	–
O-levels or equivalent	241	25.1	1.168 (0.880 – 1.551)	0.282	1.157 (0.863 – 1.552)	0.329
A-levels or equivalent	102	10.6	1.236 (0.835 – 1.832)	0.290	1.281 (0.848 – 1.935)	0.239
Further education but not degree	82	8.5	1.250 (0.812 – 1.923)	0.311	1.258 (0.803 – 1.973)	0.316
Bachelors or equivalent	115	12.0	1.426 (0.966 – 2.107)	0.074	1.382 (0.918 – 2.079)	0.121
Higher degree – Masters or PhD	45	4.7	1.200 (0.690 – 2.088)	0.519	1.211 (0.685 – 2.141)	0.509
Ethnicity					
Asian	57	5.9	1.00	–	1.00	–
Black	31	3.2	0.462 (0.212 – 1.009)	0.053	0.386 (0.172 – 0.864)	**0.021**
Mixed	18	1.9	0.671 (0.248 – 1.812)	0.431	0.494 (0.178 – 1.368)	0.175
Other	26	2.7	0.554 (0.238 – 1.291)	0.171	0.456 (0.190 – 1.091)	0.078
White	818	85.2	0.622 (0.357 – 1.084)	0.094	0.598 (0.333 – 1.072)	0.084
Missing	10	1.0	–	–	–	–

**Table 5:** Comparison of perception of information provided in results letter and satisfaction with process of results communication by letter between participants who remembered receiving ‘The SUMMIT Study: Next Steps’ booklet and those who did not.

	Overall (n = 2,014)		Participants who remembered receiving booklet (n = 1,412)		Participants who did not remember receiving booklet (n = 602)	
	Frequency (n)	%	Frequency (n)	%	Frequency (n)	%
How do you feel about the amount of information in the results letter?						
Too much information	11	0.5	7	0.5	4	0.7
Just the right amount of information	1,556	77.3	1,122	79.5	434	72.1
Not enough information	215	10.7	150	10.6	65	10.8
Can’t remember	142	7.1	85	6.0	57	9.5
N/A	90	4.5	48	3.4	42	7.0
How satisfied or dissatisfied were you with receiving your results by letter?						
Satisfied	1,668	82.8	1,195	84.6	473	78.6
Neither satisfied nor dissatisfied	199	9.9	129	9.1	70	11.6
Dissatisfied	57	2.8	40	2.8	17	2.8
Did not receive results letter	45	2.2	23	1.6	22	3.7
Can’t remember	45	2.2	25	1.8	20	3.3
