# Supplementary material for: The SUMMIT Study: Utilising a written ‘Next Steps’ information booklet to prepare participants for potential lung cancer screening results and follow-up
Source: Lung Cancer. Author manuscript; Available in PMC 2023 Oct 6. (PMC7615157; doi:10.1016/j.lungcan.2022.12.006)
Supplement: Supplementary data [file EMS188585-supplement-Supplementary_data.pdf]

YOUR RESULTS LETTER

A letter will be sent to you and your GP within three weeks of your scan. This letter will indicate one of the following outcomes.

Normal result → No sign of anything abnormal on your scan. → **Appointment at lung health check centre in approximately one year - see next page.**

Needs further tests → This means we have seen something on the scan that needs more investigation. This could include:

A nodule: We often find a small spot called a 'nodule' on the lung. In nine out of ten cases this is something harmless, but it needs to be checked out with another scan. → **You will be invited for a follow-up scan at the lung health check centre in approximately three months.**

OR

Abnormal result: This means there is something abnormal on the scan that needs more tests. It could be cancerous or it could be harmless. → **You will be invited to an appointment with a specialist doctor within two weeks.**

Other health conditions → We sometimes find other health conditions that need treatment. Most of these can be managed by your own GP and do not need urgent care. → **We will explain in the letter what we found and the next steps, for example booking an appointment with your GP.**

YOUR SUMMIT STUDY APPOINTMENTS

Not everyone will attend each of the appointments below. Most people will only attend once a year for three years.

Today → Consultation → Blood sample → Low dose CT scan

+ 3 months → Some people will need to return 3 months after their first visit to have a repeat scan

1 year from today → Consultation → Blood sample → Half will have a scan / Home

+ 3 months → Some people will need to return 3 months after their first visit to have a repeat scan

2 years from today → Consultation → Blood sample → Low dose CT scan

DON'T FORGET You will receive a £20 shopping voucher for each annual appointment you attend to thank you for your time.

WHAT CAN I DO TO REDUCE MY RISK OF LUNG HEALTH PROBLEMS?

The single best thing you can do to prevent lung cancer and other lung diseases is not smoke.
If you do smoke and would like to stop there is lots of help out there.

NHS smokefree on 0300 123 1044
or visit www.nhs.uk/smokefree

Stop Smoking London:
www.stopsmokinglondon.com
or ring 0300 123 1044 to speak with a specially trained advisor.

You can also ask your GP about local support available.

NHS

WHAT ARE THE IMMEDIATE BENEFITS OF QUITTING?

2 DAYS	Carbon monoxide cleared
3 DAYS	Breathing easier and more energy
2-12 WEEKS	Circulation improves
3-9 MONTHS	Cough and lung function improves
1 YEAR	Risk of heart attack about half that of smokers
10 YEARS	Risk of lung cancer about half that of smokers

NHS

Please keep an eye out for your LDCT results letter in the next few weeks. Of course, if you notice any new symptoms at any point, please see your GP as you normally would.

We will contact you when it is time to set up your next appointment.

Need more information before your next appointment?

For more information please call us on 0808 196 2286 or email us at uclh.summitstudy@nhs.net

Additionally, please visit our website: www.summitstudy.co.uk

NHS

THE SUMMIT STUDY

IT'S ABOUT TIME

DETECT EARLY. TREAT EARLY.
LET'S TACKLE CANCER.

NEXT STEPS

Thank you for joining the SUMMIT Study.

This booklet describes the types of results you can expect to receive from your low-dose CT (LDCT) scan and the schedule of future study visits.

GRAIL UCL
NHS University College London Hospitals NHS Foundation Trust
